# Tree Diversity Mediates the Distribution of Longhorn Beetles (Coleoptera: Cerambycidae) in a Changing Tropical Landscape (Southern Yunnan, SW China)

**DOI:** 10.1371/journal.pone.0075481

**Published:** 2013-09-19

**Authors:** Ling-Zeng Meng, Konrad Martin, Andreas Weigel, Xiao-Dong Yang

**Affiliations:** 1 Key Laboratory of Tropical Forest Ecology, Xishuangbanna Tropical Botanical Garden, Chinese Academy of Sciences, Yunnan, China; 2 University of Hohenheim, Agroecology in the Tropics and Subtropics (380b), Stuttgart, Germany; 3 Rosalia Umweltmanagement, Am Schloßgarten 6, Wernburg, Germany; York U, Canada

## Abstract

Longhorn beetles (Coleoptera : Cerambycidae) have been used to identify sites of high biological diversity and conservation value in cultivated landscapes, but were rarely studied in changing landscapes of humid tropics. This study was conducted in a region of southern Yunnan, China, which was dominated by natural rainforest until 30 years ago, but is successively transformed into commercial rubber monoculture plantations since that time. The objectives were to investigate longhorn beetle species diversity and distribution in the major land use types of this landscape and to estimate the effects of an expected expansion of rubber plantations on the longhorn beetle assemblages. The results showed that tree species diversity (181 species in total) and longhorn beetle diversity (220 species in total) were closely related with no significant differences between the tree and longhorn beetles assemblages shown by similarity distance analysis. There was a highly positive relationship between the estimated species richness of longhorn beetles and the number of tree species. Individual numbers of longhorn beetles and trees were also highly positive related at the sampling sites. Non-metric multidimensional scaling revealed that the degree of canopy coverage, succession age and tree diversity explained 78.5% of the total variation in longhorn beetle assemblage composition. Natural forest sites had significantly higher numbers of species and individuals than any other type of habitat. Although young rubber plantations bear the highest longhorn beetle diversity outside forests (half of the total number of longhorn beetle species recorded in total), they can not provide permanent habitats for most of these species, because they develop into closed canopy plantations with less suitable habitat conditions. Therefore, along with an expected expansion of rubber cultivation which largely proceeds at the expense of forest areas, the habitat conditions for longhorn beetles in this region might decrease dramatically in future.

## Introduction

Longhorn beetles (Coleoptera : Cerambycidae) have been used to identify sites of high biological diversity and conservation value in cultivated landscapes which are usually composed of heterogeneous mosaics of different land use [1], [2], [3]. Longhorn beetles almost exclusively feed on living, dying or dead woody plants in the larval stage. Relationships between longhorn beetles and host plants are often quite specific, but there is a great range in the breadth of host tree species that may used by the larvae of different species [4]. Longhorn beetles can play an important role in the decomposition of dead wood and therefore were also considered as “ecosystem engineers” [5]. Furthermore, many longhorn beetle adults visit flowers to feed on nectar and/or pollen and therefore act as pollinators.

Longhorn beetle diversity and distribution was shown to be affected by forest management practices [6], [7], [8], [9], [10], invasive tree species [11], habitat destruction and degradation [12], [13], habitat fragmentation [14], environmental gradients [15], disturbances of fire, drought and windstorm [16], [17], [18], [19], [20], [21], spatial heterogeneity [22] and effects of host species preferences [23]. Conclusions drawn from those studies are that most longhorn beetle species are concentrated on undisturbed or primary forest, whereas secondary forest and artificial plantations support less species. Furthermore, increasing intensification and disturbance tends to reduce specialist longhorn beetle species and to homogenize the beetle assemblages between the various habitat types of a landscape. Most studies were conducted in temperate regions, where the original vegetation has disappeared or been strongly modified in the course of a usually long history of land cultivation. However, very little research has been done to analyze the effects of land use change on beetle assemblages in relatively young cultivated landscapes of tropical rainforest regions (but see [24] for Mexico).

This study was conducted in the tropical landscape of southern Yunnan Province, China. This region is part of the ‘Indo-Burma hotspot’, one of the 34 global hotspots exceptionally rich in biodiversity [25]. The specific study area represents a tributary valley of the Mekong River. There, traditional land use systems are irrigated rice fields along the river courses and shifting cultivation systems on the slopes, but the largest proportion of the land area was covered with primary and secondary forest until about 30 years ago. Since then, large areas of forest have been, and still are, successively transformed into commercial rubber (*Hevea brasiliensis*) monoculture plantations. The predominant habitat types of the investigated landscape therefore include natural forest plots, open shrubland and grassland and agricultural fields as well as rubber plantations of different age. This land use pattern is representative of the development of tropical southern Yunnan. Detailed data from a typical subregion (Xishuangbanna Prefecture) showed that between 1998 and 2006, rubber plantations increased from 12% of the total land coverage to 46%, whereas forested areas dropped from 49 to 28% [26]. Tropical seasonal rainforest was the type of land most affected by the expansion of rubber plantations [27].

The objective of this study was to investigate the longhorn beetle species diversity and distribution in this fragmented landscape in relation to land use type and to assess the response of the longhorn beetle guilds to the recent changes in land use. Due to the importance of woody plants as resources for longhorn beetles, the land use types were characterized by their tree species inventory, which largely corresponds with the successional stage of the predominant land use types, including rice field fallows, grassland and shrubland, young and old rubber plantations and natural forest plots. These data are used to estimate the effects on longhorn beetle diversity and distribution as a consequence of the expected increase of rubber plantations, which is related to the loss and reduction of forest cover and the increasing fragmentation of the landscape. Based on the idea that land use changes by human beings affect the species richness and composition in longhorn beetles, we hypothesis that some specialist species will be more sensitive than others to forest coverage decreased. We also expect that artificial rubber plantation at young ages especially those closing to forest patches which were mixed with some young tree species can be served as a temporary suitable habitat for some generalist species.

## Materials and Methods

### Ethics Statement

The Naban River Watershed National Natural Reserve (NRWNNR) provided the permission for the field studies.

### Study area and Sampling Localities

The study was carried out in the Naban River valley (ca. 11.000 ha) within the Naban River Watershed National Nature Reserve (NRWNNR) in Xishuangbanna, southern Yunnan province, south-west China (22° 10′ N and 100° 38′ E). The region represents the northernmost part of the humid tropics in Asia with a climate influenced by Monsoon and three distinct seasons: cool-dry (October-January, with the lowest monthly temperature of 15°C in December), hot-dry (February-April, with the highest monthly temperature of 25°C in April) and a rainy season (May-September) with annual precipitation of almost 1600 mm. The natural vegetation of the study region is tropical rainforest, consisting of different types of evergreen and seasonal forest depends on topography and elevation [28], [29]. Secondary and primary forest plots and fragments are widespread in the study area, but most cultivated land is covered by rubber plantations. Valley bottoms are dominated by rice fields, and there are various fruit and vegetable crops around the small villages. Shrubland and grassland areas are found along the slopes. To represent the most typical habitat types of this landscape, we selected 13 sampling localities including forest plots, rubber plantations, fallows and open lands (Table S1). Further descriptions are given in Table 1.

**Table 1:** Overview and description of the 13 sampling sites in the study area of the Naban River valley within Naban River Watershed National Nature Reserve (NRWNNR).

Location	Site code	Site descriptions
**Forest**		
Mandian	MD-FO	Primary forest, closed canopy 35 m high
Naban	NB-FO	Secondary forest, closed canopy 35 m high
Anmaxinzhai	AM-FO	Secondary forest, closed canopy 35 m high
Guomenshan	GMS-FO	Primary forest, closed canopy 35 m high
**Rubber plantations**		
Mandian	MD-RU	5 years, trees 7 m high, open canopy
Naban	NB-RU	8 years, trees 12 m high, open canopy
Anmaxinzhai	AM-RU	20 years, trees 20 m high, closed canopy
Shiyidui	SYU-RU	40 years, trees 30 m high, closed canopy
**Open land**		
Naban	NB-OP	Forest clearfell between NB-FO and NB-RU
Anmaxinzhai	AM-OP	Grassland on a ridge
Guomenshan	GMS-OP	Shrubland succession
**Fallow**		
Mandian	MD-FA	Rice field fallow
Guomenshan	GMS-FA	Rice field fallow

### Field Methods

Beetle sampling was carried out by using a combined trap system including bowl traps and Malaise traps (3.5×2.0×1.5 m, length×width×height) [30] at all sites, and aerial collectors in the canopy area of trees in forests and in rubber plantations. Bowl traps were plastic pots with a diameter of 35 cm and a depth of 15 cm put on the soil surface, one third height filled with a mixture of liquid of blue colored anti-freeze (ethanol-glycol). At each site, one yellow bowl trap was arranged at a distance of ca. 5 m from one side of Malaise trap, and a red bowl trap was established at the other side with a same distance. Malaise traps had two separate collection ports and were installed in directions that cover the typical land use type of the trap site. Aerial collectors were constructed of two pieces of transparent plastic plates (50×30 cm, height×width) which were arranged crosswise and fixed upon a red plastic bowl of 30 cm in diameter. These traps were installed on canopy tree branches using ropes. The collecting bottles of the Malaise traps and the bowls of the aerial collectors were also filled with a mixture of liquid of blue colored anti-freeze (ethanol-glycol).

Traps collections were conducted in different seasonal periods covering (a) the beginning of the rainy season (May-July 2008), (b) the beginning of the cool-dry season (September-November 2008), and (c) the transition period from the hot-dry to the rainy season (March-June 2009). At all sites, traps were emptied every 10 days during the collecting periods (with few exceptions where traps were destroyed or collection was impossible due to heavy rains). The beetle specimens were preserved in 70% ethanol for further identification to species level. Data analyses are based on numbers of species and individuals combined from all traps and trap types per site and the total counts from all collecting periods. Voucher specimen of the collected beetles are kept at the National Zoological Museum of China, Institute of Zoology, CAS, Beijing.

Vascular plant species inventories from each of the 13 trap sites were recorded in March 2009. At the four natural forest and four rubber plantation sites, four 20×20 m^2^ plots were established around the trap locations to record the numbers of tree and liana species. Other plant species including young trees (representing the groundcover vegetation <2 m) in the plots were recorded from four 5×5 m^2^ subplots within each of the four large plots. Total species numbers of the four small and the four large plots were used for further calculations. In the three open land and two fallow sites, records from all four 20×20 m^2^ plots per site provided the plant species numbers used for calculations. Voucher specimens of the recorded and identified plant species were deposited at the Herbarium of the Xishuangbanna Tropical Botanical Garden (XTBG), CAS, Yunnan, China.

### Data Analysis

Cluster analyses through stratigraphically constrained clustering were performed to identify quantitatively similar groups of tree species (including young trees but without shrub and liana) and longhorn beetle assemblages among the different sites and habitats using Bray-Curtis index of similarity. Among the algorithms for hierarchical clustering, we selected the unweighted pair-group method using averages (UPGMA) which is conventionally used in ecology [31], [32]. One-way ANOSIM (analysis of similarities) global tests were then applied to test for differences between subgroups produced by the cluster analysis. The abundance data of tree and longhorn beetle were log normalized through log_10_(X+1) transformation to bring out category features of original data and downweight those a few very dominant taxa. Differences in total species numbers and abundances of longhorn beetles between the subgroups were compared by the Mann-Whitney *U*-test using Minitab 15.0 software (Minitab Inc. State College PA, USA). This non-parametric test was applied because the assumptions of homogeneity of variances and normality (tested with the Shapiro-Wilk normality test) were not met according to [33]. Furthermore, we used a one-way ANOVA to test whether the Bray-Curtis similarity distance indices among the 13 sampling sites produced by tree and longhorn beetle data assemblages were different from each other.

Non-metric multidimensional scaling (NMS; [34]) using the Bray–Curtis index for abundance data was applied to display and test for differences in longhorn beetle assemblage composition across the land use types. This ordination score was performed with PC-ORD software [35] with the following parameters employed in the NMS procedure: Sorensen distance measure; a maximum number of 500 iterations; random starting coordinates; 100 runs with real data; step down in dimensionality (initial step length = 0.2); 100 runs with randomized data. A total of 12 vegetation and land use variables after log transformation were included in the NMS analysis to test their effects on the longhorn beetle assemblages. Two variables that refer to number of tree species and individuals, respectively, were included. Further 5 variables refer to plant species richness, including the total number of vascular plant species per site and the species numbers of different life forms, i.e. grasses, forbs (non-woody plants other than grasses), and lianas. The remaining variables are the degree of tree canopy cover and the degree of ground vegetation cover, the maximum vegetation height, the successional age of the study site (years after establishment of the present vegetation or age of the trees), and the discrimination between four categories of land use type, represented by rice field fallows, early successions (forest clear fell, grassland, scanty shrubland), rubber plantations and natural forest (Table 1). Correlations between the ordination and the environmental variables were calculated with the Pearson coefficient. Indicator Species Analysis based on the combined values of relative abundance and relative frequency of species [36] was used to identify longhorn beetle species affiliated with specific land use types. The indicator value of each of the recorded species was calculated with PC-ORD software [35] using 4999 runs in a Monte Carlo test considering values at *P*<0.05.

The Chao 1 estimator was used to estimate total species richness of longhorn beetles per site. This estimator is the sum of the observed number of species and the quotient *a* ^2^/2*b*, where *a* and *b* equal the number of species represented by one (singletons) and two (doubletons) individuals, respectively. Calculations were conducted using the software package EstimateS (Version 8.2.0; [37]). To examine the relationship between Chao 1 estimated species richness of longhorn beetles and the variables of tree species and individuals at sampling location, these were included in the NMS analysis, using polynomial regression and Spearman’s Rho non-parametric correlation.

## Results

**Figure 1:**
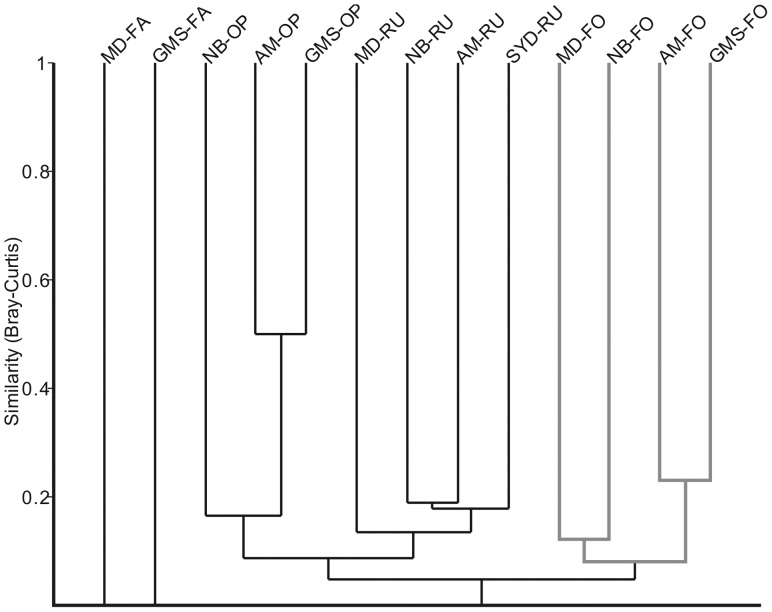
Quantitative similarity cluster analysis of the tree species at the sampling localities (site codes see Table 1), generated from the Bray-Curtis index using UPGMA through stratigraphically constrained clustering. The dendrogram shows four subgroups clearly classified by habitat categories.

A total of 1434 tree individuals representing 181 species were recorded across all study sites (Table S2). The cluster analysis dendrogram based on the quantitative similarities of the tree inventories is shown in Fig. 1. As supported by global one-way ANOSIM tests, meaningful differences between assemblages occurred at a similarity of almost 0.1 and generated 4 subgroups (global *R* = 0.622; *P*<0.0001). The dendrogram shows two major subgroups which are clearly distinguished by land use categories. Subgroup 1 includes all four mature forest sites. Subgroup 2 represents the four rubber plantation sites of different age (2a) and three open land sites (2b). The two fallow sites clearly diverge from the other groups due to their extremely low presence of trees.

**Figure 2:**
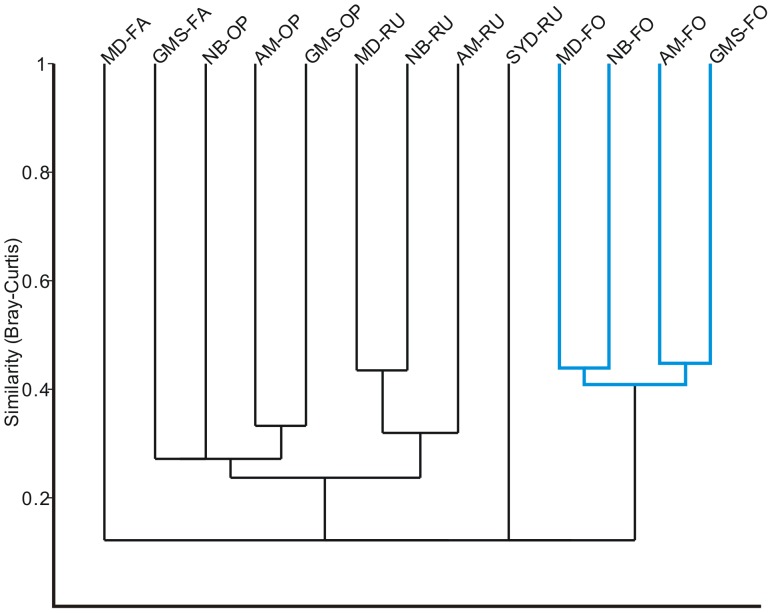
Quantitative similarity cluster analysis of the longhorn beetles at the sampling localities (site codes see Table 1), generated from the Bray-Curtis index using UPGMA through stratigraphically constrained clustering. The dendrogram shows three subgroups clearly classified by habitat categories.

A total of 1404 specimens representing 220 longhorn beetle species were recorded across all study sites and recording periods. Consistent with the highest tree species diversity, more than half (780) of all individuals were recorded from forest sites (Table S3). Meaningful differences between communities occurred at a similarity of about 0.25 and generated three main subgroups (global *R* = 0.4093; *P*<0.01; Fig. 2). Subgroup 1 is composed of the four forest sites. Subgroup 2 is represented by three of the four rubber plantation sites. Subgroup 3 includes three open land sites and one fallow site. Another fallow site and the youngest rubber plantation (5 years) diverge from these subgroups. The Bray-Curtis similarity distance index among 13 sampling sites showed no significant differences between the tree and longhorn beetles assemblages (one-way ANOVA, *F* = 2.864, df = 1,181, *P* = 0.092).

**Figure 3:**
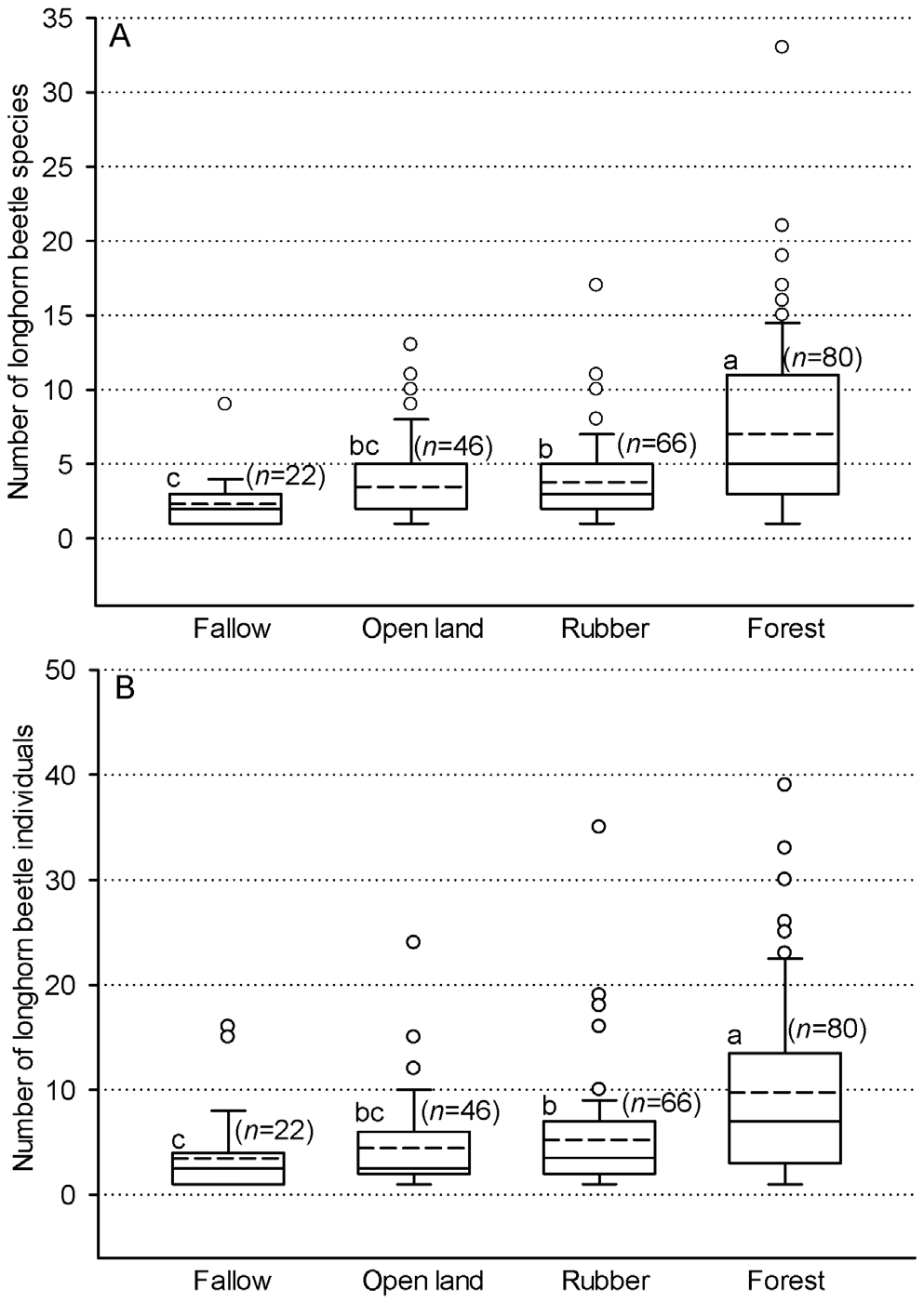
Number of (A) species and (B) individuals of longhorn beetles in the four subgroup produced by habitat categories. Box and whisker plots illustrate the 5th, 25th, 50th (median), 75th, and 95th percentiles, and the means as the dashed line. Different letters indicate significant differences (*P*<0.05; Mann-Whitney *U*-Test). Small circle indicate outliners.

Based on the analysis of the a priori selected habitat types, Fig. 3 shows that forest sites had the highest and fallows had the lowest mean value of both species and individuals. Rubber plantations and open land sites showed intermediate values with no significant differences between each other. The mean number of species and individuals was significantly different comparing all habitat types (*P*<0.001; Mann-Whitney *U*-Test).

**Figure 4:**
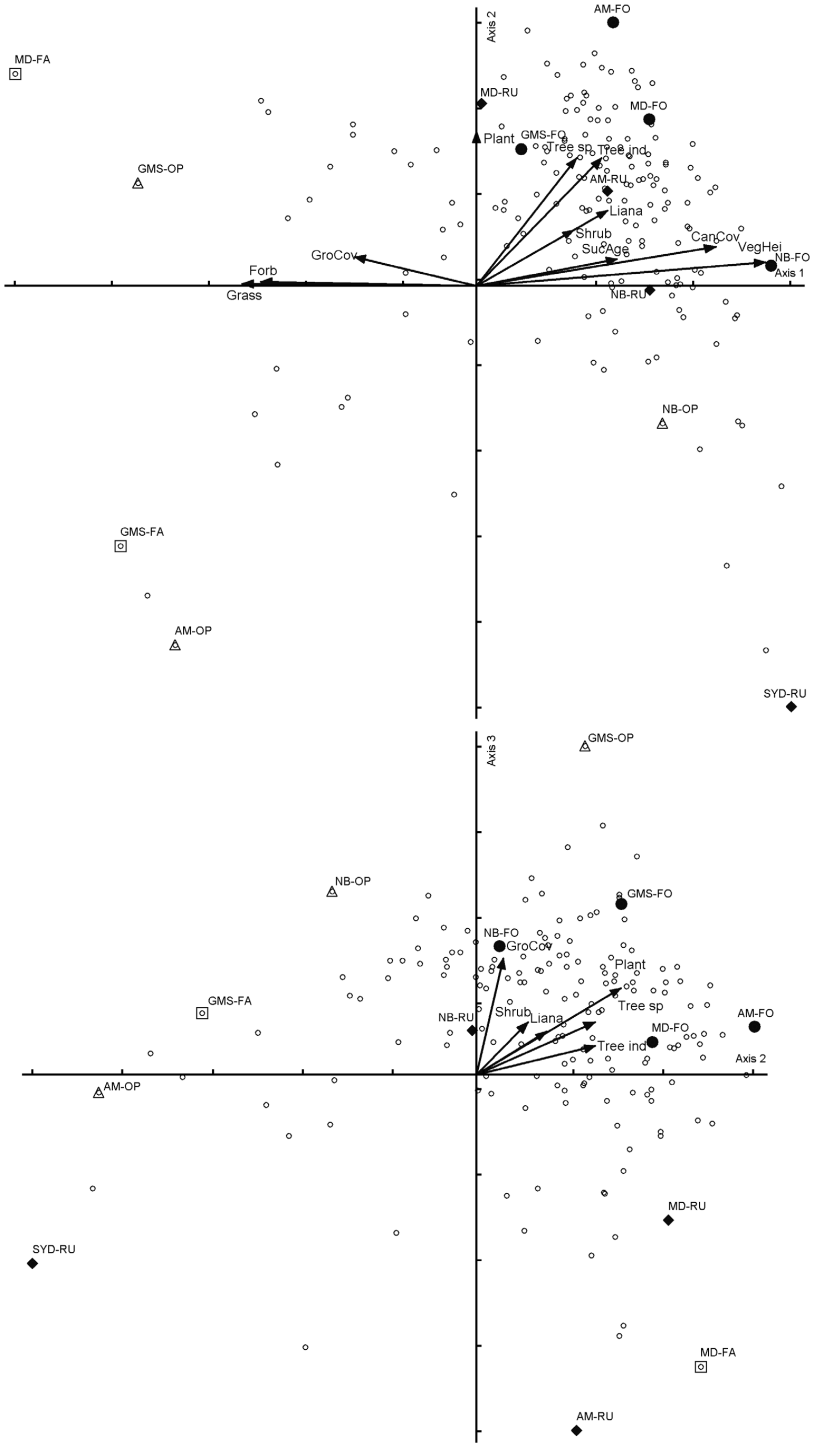
NMS ordination of longhorn beetle assemblages at the 13 sample sites: (♦) rubber plantations; (△) grassland and shrubland; (□) rice field fallows; (•) forest. Blue small circle means longhorn beetle species. Variables with Pearson correlation coefficient at *P*<0.05 are shown. Cumulative variation in the original dataset explained by ordination is 78.5% (Axis 1 = 41.0%, Axis 2 = 19.2%, Axis 3 = 18.4%, Final stress = 9.58, Final instability = 0.00001).

The NMS ordination of the longhorn beetle assemblages represented 78.5% of the variation with a recommended three dimensional solution (Fig. 4) at a final stress of 9.58. Axis 1 accounts for 41.0% of this variation while axes 2 and 3 represent 19.2% and 18.4%, respectively. All environmental variables except the land use category showed significant effects at *P*<0.05 (Pearson correlation coefficient) for longhorn beetles (Fig. 4). Axis 1 was not only positively related with vegetation height, canopy coverage and succession age, but also with the plant variables referring to the number of tree species and individuals and also to the numbers of shrubs and lianas. Contrary to this, Axis 1 was negatively related with the ground coverage and number of grass and forb species at the sampling sites. Six longhorn beetle species affiliated with forest sites with indicator values at *P*<0.05 were identified. No indicator species affiliated with the other three land use types were found (see Table S3).

**Figure 5:**
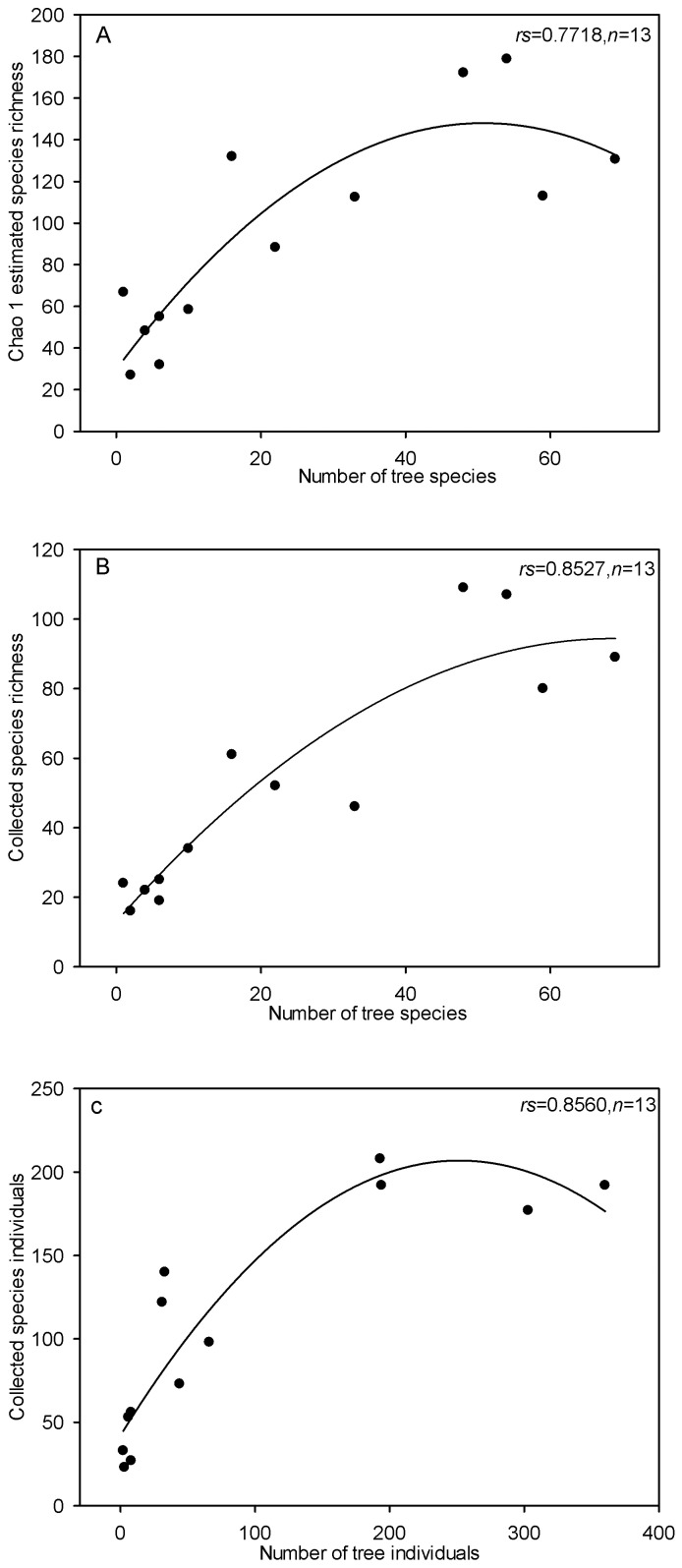
The polynomial regression between the diversity index of trees and longhorn beetles at 13 sampling location within Naban River Watershed National Nature Reserve. (A) Number of tree species and estimated Chao1 species richness of longhorn beetles; (B) Number of tree species and collected species richness of longhorn beetles; (C) Number of tree individuals and collected species individuals of longhorn beetles.

There was a highly positive relationship between the estimated species richness of longhorn beetles and the number of tree species at the sampling sites (*rs* = 0.7718; *P*<0.001) (Fig. 5a). The species richness of longhorn beetles and the number of tree species also showed a highly positive relationship between each other (*rs* = 0.8527; *P*<0.001) (Fig. 5b). Furthermore, longhorn individual numbers was also highly positive related to the number of tree individuals at the sampling sites (*rs* = 0.8560; *P*<0.001) (Fig. 5c). The polynomial regression between the diversity index of trees and longhorn beetles at the sampling sites showed a highly positive relationship (*P*<0.01).

## Discussion

The overall results on longhorn beetle species richness and abundance in the different habitat types of the study landscape clearly indicate that only one land use type, natural forest, possesses a degree of uniqueness in species diversity. The highest total numbers of species and individuals were recorded from the forest sites at which 780 individuals represented 193 species, from 220 species recorded overall. Furthermore, longhorn beetle species numbers at the forest sites were related to tree species diversity at the respective sites. However, only 6 forest habitat specialist species were identified. The number of forest specialists might indeed be higher than the estimated species number through the Chao estimator, because most of the species were only represented by few individuals which were not enough to show statistically significant results. For example, from the 193 species recorded from forest sites, 166 species were only represented with numbers between one and 6 individuals. Although these species were not denoted as indicator species, most of them are probably forest specialists, but they are merely rare.

The positive relationships between the numbers of longhorn beetle species and individuals on the one hand and the number of tree species and individuals on the other clearly indicate the importance of forest diversity for the longhorn beetle assemblages of the study landscape. Tree diversity may also indirectly reflect the availability of dead and decaying wood for various specialists. The quantity of this resource was not analyzed explicitly in the present study, but was found to be of high importance for saproxylic beetles in studies of northern temperate and boreal forests [38], [39].

The highest number of longhorn beetle species outside forest habitats were recorded from rubber plantations (109 species, 344 individuals), with higher numbers in young plantations (5 and 8 years) than in older stands (20 and 40 years). It can be assumed that most of the species recorded there originate from natural forest sites and temporarily colonized rubber plantations due to the structural similarity of these plantations with forest. Most longhorn beetle species including the most common ones (*Chlorophorus arciferus*, *Pterolophia annulata* and *Xylotrechus buqueti*) were recorded from both forest and rubber plantations. However, for a few species such as *Nupserha nigriceps*, young rubber plantations might represent a suitable habitat because they were most common in this type of habitat and rare in forest sites. Young rubber plantations established after the clear felling of natural forest have open canopies and exhibit a rich undergrowth vegetation with regrown young forest trees and dead wood that remained from the destroyed forest. These conditions might explain the higher longhorn beetle diversity in young compared to old closed rubber stands. The latter only show a poor undergrowth vegetation, because most of the plants occurring in young plantations became shaded out after the closing of the rubber tree canopy. Similarly, a study conducted in Japan [40] showed that in different in types of plantation forests (broad leaf and conifer), the species richness of longhorn beetles was highest in young stands and decreased with the age of the stand. Possible causes of the high species richness in young stands stated by these authors include the availability of large amounts of coarse wood debris and flowers, which are resources for oviposition and nutrition for adults, respectively. The same study showed that longhorn beetle diversity was generally lower in conifer stands than in broad-leaved stands, and almost all species that occurred in conifer stands were also collected in broad-leaved stands. Another study conducted in Japan [6] showed that species richness of longhorn beetles was lower in second-growth forests and conifer plantations than in old-growth forests. These results are principally consistent with the findings of the present study referring to the longhorn beetle diversity patterns of forest, young rubber plantations and old rubber plantations, which decreases in this sequence. Although young rubber plantations bear the highest longhorn beetle diversity outside forests (almost half of total number of longhorn beetle species recorded in this study), they can not be considered as alternative habitats for most of the longhorn beetle species originating from forest, because they develop into closed canopy plantations with less suitable habitat conditions. This stage is approximately reached 20 years after planting. Currently, most of the rubber plantations in the study area are less than 20 years old, but will reach an age of about 40 years before the latex productivity declines. Then, the plantations will be clear felled for starting a new plantation cycle with young trees. Therefore, along with an expected expansion of rubber cultivation which largely proceeds at the expense of forest areas, the habitat conditions for longhorn beetles might decrease dramatically in future. Nonetheless, effects of rubber cultivation on longhorn beetles could be mitigated by preserving diverse natural forest plots and in addition, by a management of the rotation cycles of rubber plantations that allows the steady presence of young plantation stages. As recommended in various studies [10], [38], [41], the leaving of old trees, snags and dead wood in clear-cuts could also contribute to the preservation of longhorn beetles in newly established rubber plantations.

Similar to rubber plantations, most longhorn beetle species recorded from fallow, grassland and shrubland sites were also found in forests. The relatively low species and individual numbers from these habitat types might indicate that most species appeared there incidentally, reflecting the mobility of species which are able to move within the landscape. Overall, the highest species richness is clearly concentrated on the forest plots of the study landscape. On the other hand, a study from a temperate landscape of Switzerland [42] indicates that ecotones with a gradual transition from open land into the forest provide an important habitat for longhorn beetles, and the authors conclude that many of the so-called forest species are in fact forest edge species. They argue that additional resources such as pollen, sunlight and heat are necessary to meet the demands of many adult longhorn beetles, while in the forest interior a sufficient amount of dead wood as a substrate for larval development may be available. However, besides of the differences in climate and overall species diversity, these two landscapes differ in their land use history. The research area of the present study is characterized by recent and extreme changes through rubber cultivation, whereas many cultivated landscapes of temperate Europe have developed over hundreds or thousands of years and now provide habitats for species originating from other (warmer) regions. We therefore assume that the largest portion of the recorded longhorn beetle assemblage in the tropical landscape of the present study originates from the local natural forest, and that with few exceptions (such as *Nupserha nigriceps*) the large majority of the species can not benefit from wood provided by rubber trees and from the creation of open habitats. This, however, was not the case in certain other groups of insects recorded from the same region. For example, Meng et al. [43] found that the species richness and densities of ground beetles (Carabidae) were significantly higher in open habitats and rice field fallows than in other habitat types of this landscape, including forest, and are probably dominated by species originating from other regions with natural open vegetation. Furthermore, rice field fallows, early natural successions and natural forest each possess a degree of uniqueness in ground beetle species composition. Total variation in ground beetle assemblage composition was explained by the degree of vegetational openness and the groundcover plant species diversity. That is, grass and forb species showed to be the most important plant life types accounting for ground beetle distribution, and not trees. Compared to the ground beetles, longhorn beetles therefore seem to be more suitable indicators for the ecological state of landscapes in tropical forest regions.

## Supporting Information

Table S1 Habitat characteristics of the 13 sampling sites and the environmental variables and the environmental variables used in the NMS analysis: total number of vascular plant species (Plant) and species of different life forms; percentage of ground vegetation cover (GroCov) and canopy cover (CanCov); the successional age of the site or age of the trees (SucAge), vegetation height (VegHei) and four categories of land use.
(DOCX)

Table S2 List of tree species and numbers of individuals recorded from the 13 study localities compiled by habitat types. Abbreviation code FA, OP, RU and FO means rice fallow, open land, rubber plantation and forest respectively.
(DOCX)

Table S3 List of longhorn beetle species and numbers of individuals recorded from the 13 study localities compiled by habitat types (*means indicator species). Abbreviation code FA, OP, RU and FO means rice fallow, open land, rubber plantation and forest respectively.
(DOCX)
