# Correction: Tree Diversity Mediates the Distribution of Longhorn Beetles (Coleoptera: Cerambycidae) in a Changing Tropical Landscape (Southern Yunnan, SW China)

**DOI:** 10.1371/annotation/bfe068c2-f5ce-4bc6-83d2-8fe47861ac03

**Published:** 2013-11-14

**Authors:** Ling-Zeng Meng, Konrad Martin, Andreas Weigel, Xiao-Dong Yang

In Table 1, the abbreviation of plantations sites for Shiyidui was listed as “SYU-RU”, it should read as “SYD-RU.”

In Figure 1, the cluster of four forest sites lost the appropriate coloring during the production process. Please see the corrected Figure 1 here: 

**Figure:**
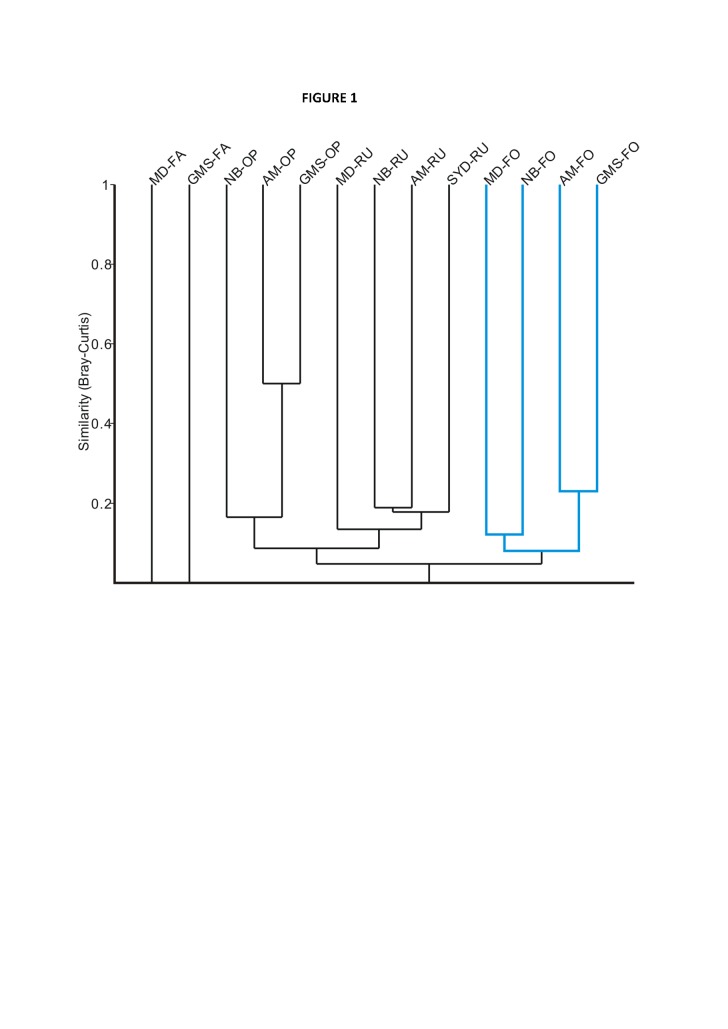


In the second paragraph of the Results section, the sentence “Another fallow site and the youngest rubber plantation (5 years)” should read “Another fallow site and the oldest rubber plantation (40 years).”

