# Supplementary material for: Tree Diversity Mediates the Distribution of Longhorn Beetles (Coleoptera: Cerambycidae) in a Changing Tropical Landscape (Southern Yunnan, SW China)
Source: PLoS One. 2013 Sep 19;8(9):e75481. doi: 10.1371/journal.pone.0075481 (PMC3777904; doi:10.1371/journal.pone.0075481)
Supplement: Table S1 — Habitat characteristics of the 13 sampling sites and the environmental variables and the environmental variables used in the NMS analysis: total number of vascular plant species (Plant) and species of different life forms; percentage of ground vegetation cover (GroCov) and canopy cover (CanCov); the successional age of the site or age of the trees (SucAge), vegetation height (VegHei) and four categories of land use. (DOCX) [file pone.0075481.s001.docx]

**Supporting Information**

**Table S1** Habitat characteristics of the 13 sampling sites and the environmental variables and the environmental variables used in the NMS analysis: total number of vascular plant species (Plant) and species of different life forms; percentage of ground vegetation cover (GroCov) and canopy cover (CanCov); the successional age of the site or age of the trees (SucAge), vegetation height (VegHei) and four categories of land use.

| Study site | No. of plant species and tree individuals | | | | | | | GroCov | CanCov | SucAge | VegHei | Land |
| --- | --- | --- | --- | --- | --- | --- | --- | --- | --- | --- | --- | --- |
| (Code) | Plant | Grass | Forb | Liana | Shrub | Tree sp | Tree indi | (%) | (%) | (years) | (m) | use |
| Forest (MD-FO) | 111 | 7 | 14 | 10 | 26 | 54 | 303 | 50 | 95 | 80 | 30.3 | 3 |
| Forest (NB-FO) | 137 | 2 | 17 | 15 | 34 | 69 | 360 | 68 | 90 | 70 | 33.4 | 3 |
| Forest (AM-FO) | 117 | 10 | 14 | 10 | 24 | 59 | 194 | 73 | 95 | 80 | 21.5 | 3 |
| Forest (GMS-FO) | 111 | 18 | 17 | 10 | 18 | 48 | 193 | 75 | 85 | 60 | 28.5 | 3 |
| Rubber (MD-RU) | 74 | 16 | 14 | 5 | 6 | 33 | 66 | 30 | 75 | 4 | 10.13 | 2 |
| Rubber (NB-RU) | 62 | 12 | 21 | 2 | 5 | 22 | 33 | 21 | 87 | 8 | 11.75 | 2 |
| Rubber (AM-RU) | 45 | 10 | 10 | 4 | 11 | 10 | 44 | 12 | 94 | 20 | 20 | 2 |
| Rubber (SYD-RU) | 17 | 5 | 7 | 1 | 3 | 1 | 2 | 5 | 95 | 45 | 30 | 2 |
| Open land (NB-OP) | 55 | 8 | 19 | 3 | 9 | 16 | 31 | 85 | 1 | 2 | 2.5 | 4 |
| Open land (AM-OP) | 57 | 18 | 18 | 3 | 12 | 6 | 8 | 75 | 1 | 5 | 1.2 | 4 |
| Open land (GMS-OP) | 66 | 20 | 23 | 3 | 14 | 6 | 8 | 90 | 1 | 25 | 1.2 | 4 |
| Rice field (MD-FA) | 54 | 16 | 35 | 0 | 1 | 2 | 3 | 95 | 1 | 1 | 0.7 | 1 |
| Rice field (GMS-FA) | 64 | 22 | 38 | 0 | 0 | 4 | 6 | 97 | 1 | 1 | 0.6 | 1 |
