# Supplementary material for: Tree Diversity Mediates the Distribution of Longhorn Beetles (Coleoptera: Cerambycidae) in a Changing Tropical Landscape (Southern Yunnan, SW China)
Source: PLoS One. 2013 Sep 19;8(9):e75481. doi: 10.1371/journal.pone.0075481 (PMC3777904; doi:10.1371/journal.pone.0075481)
Supplement: Table S2 — List of tree species and numbers of individuals recorded from the 13 study localities compiled by habitat types. Abbreviation code FA, OP, RU and FO means rice fallow, open land, rubber plantation and forest respectively. (DOCX) [file pone.0075481.s002.docx]

**Supporting Information**

**Table S2** List of tree species and numbers of individuals recorded from the 13 study localities compiled by habitat types. Abbreviation code FA, OP, RU and FO means rice fallow, open land, rubber plantation and forest respectively.

| Species | FA | OP | RU(5-8y) | RU(20-40y) | FO |
| --- | --- | --- | --- | --- | --- |
| *Actinodaphne henryi* | 0 | 0 | 0 | 0 | 4 |
| *Actinodaphne obovata* | 0 | 0 | 0 | 0 | 1 |
| *Aglaia abbreviata* | 0 | 0 | 0 | 0 | 2 |
| *Aglaia perviridis* | 0 | 0 | 0 | 0 | 4 |
| *Aidia yunnanensis* | 0 | 0 | 0 | 0 | 16 |
| *Alangium kurzii* | 0 | 0 | 0 | 0 | 2 |
| *Albizia chinensis* | 0 | 0 | 0 | 0 | 1 |
| *Albizia odoratissima* | 0 | 0 | 1 | 1 | 4 |
| *Alseodaphne andersonii* | 0 | 0 | 0 | 0 | 2 |
| *Alstonia rostrata* | 0 | 0 | 0 | 0 | 2 |
| *Alstonia scholaris* | 0 | 0 | 0 | 0 | 1 |
| *Amoora yunnanensis* | 0 | 0 | 0 | 0 | 2 |
| *Antidesma montanum* | 0 | 0 | 0 | 0 | 8 |
| *Aphananthe cuspidata* | 0 | 0 | 0 | 0 | 1 |
| *Apodytes dimidiata* | 0 | 0 | 1 | 0 | 4 |
| *Aporusa dioica* | 0 | 5 | 3 | 0 | 11 |
| *Aporusa planchoniana* | 0 | 0 | 0 | 0 | 7 |
| *Aporusa villosa* | 0 | 0 | 0 | 0 | 3 |
| *Aporusa yunnanensis* | 0 | 0 | 3 | 26 | 10 |
| *Ardisia solanacea* | 0 | 0 | 0 | 0 | 1 |
| *Ardisia thyrsiflora* | 0 | 0 | 0 | 0 | 2 |
| *Artocarpus nitidus subsp.* | 0 | 0 | 0 | 0 | 1 |
| *Artocarpus pithecogallus* | 0 | 1 | 0 | 0 | 3 |
| *Arytera littoralis* | 0 | 0 | 0 | 0 | 8 |
| *Baccaurea ramiflora* | 0 | 1 | 1 | 0 | 7 |
| *Bauhinia variegata* | 0 | 0 | 0 | 0 | 14 |
| *Beilschmiedia fasciata* | 0 | 0 | 0 | 0 | 2 |
| *Beilschmiedia yunnanensis* | 0 | 0 | 0 | 0 | 56 |
| *Betula alnoides* | 0 | 0 | 0 | 0 | 1 |
| *Bischofia javanica* | 0 | 0 | 1 | 1 | 6 |
| *Bombax ceiba* | 0 | 0 | 1 | 0 | 0 |
| *Broussonetia papyrifera* | 0 | 1 | 0 | 0 | 0 |
| *Callicarpa arborea* | 0 | 0 | 0 | 0 | 3 |
| *Canarium subulatum* | 0 | 0 | 0 | 0 | 1 |
| *Canarium tonkinense* | 0 | 0 | 0 | 0 | 9 |
| *Canthium simile* | 0 | 0 | 0 | 0 | 1 |
| *Carallia brachiata* | 0 | 0 | 0 | 0 | 1 |
| *Carallia diplopetala* | 0 | 0 | 0 | 0 | 1 |
| *Casearia graveolens* | 0 | 0 | 1 | 0 | 0 |
| *Castanopsis argyrophylla* | 0 | 0 | 0 | 0 | 3 |
| *Castanopsis ceratacantha* | 0 | 0 | 0 | 0 | 4 |
| *Castanopsis fleuryi* | 0 | 0 | 0 | 0 | 22 |
| *Castanopsis hystrix* | 0 | 0 | 0 | 0 | 25 |
| *Castanopsis indica* | 0 | 0 | 0 | 0 | 1 |
| *Castanopsis mekongensis* | 0 | 0 | 0 | 0 | 20 |
| *Celtis biondii* | 0 | 0 | 1 | 0 | 2 |
| *Chassalia curviflora* | 0 | 0 | 0 | 0 | 16 |
| *Chisocheton cumingianus* | 0 | 0 | 0 | 0 | 5 |
| *Chukrasia tabularis* | 0 | 0 | 1 | 0 | 0 |
| *Cinnamomum bejolghota* | 0 | 0 | 1 | 0 | 1 |
| *Citrus maxima* | 0 | 4 | 1 | 0 | 16 |
| *Cleidion brevipetiolatum* | 0 | 0 | 0 | 0 | 1 |
| *Colebrookea oppositifolia* | 0 | 0 | 0 | 0 | 2 |
| *Colona floribunda* | 0 | 1 | 0 | 0 | 30 |
| *Cratoxylum cochinchinense* | 0 | 0 | 3 | 0 | 3 |
| *Cryptocarya yunnanensis* | 0 | 0 | 0 | 0 | 7 |
| *Cyclobalanopsis delavayi* | 0 | 0 | 0 | 0 | 2 |
| *Cyclobalanopsis kerrii* | 0 | 0 | 0 | 0 | 1 |
| *Cylindrokelupha alternifoliolata* | 0 | 0 | 0 | 0 | 3 |
| *Cylindrokelupha balansae* | 0 | 0 | 0 | 0 | 2 |
| *Cylindrokelupha yunnanensis* | 0 | 0 | 0 | 0 | 1 |
| *Dalbergia fusca* | 0 | 0 | 2 | 0 | 3 |
| *Dalbergia obtusifolia* | 0 | 0 | 0 | 0 | 1 |
| *Decaspermum fruticosum* | 0 | 0 | 1 | 0 | 1 |
| *Desmodium zonatum* | 1 | 0 | 0 | 0 | 0 |
| *Dichapetalum gelonioides* | 0 | 0 | 0 | 0 | 4 |
| *Dicranopteris ampla* | 0 | 0 | 1 | 0 | 0 |
| *Diplospora mollissima* | 0 | 0 | 0 | 0 | 2 |
| *Drypetes hoaensis Gagnep.* | 0 | 0 | 0 | 0 | 2 |
| *Drypetes sp.* | 0 | 0 | 0 | 0 | 1 |
| *Elaeocarpus apiculatus* | 0 | 0 | 0 | 3 | 0 |
| *Elaeocarpus austroyunnanensis* | 0 | 0 | 1 | 0 | 0 |
| *Elaeocarpus prunifolioides* | 0 | 1 | 0 | 0 | 1 |
| *Elaeocarpus varunua* | 0 | 0 | 0 | 0 | 1 |
| *Engelhardia spicata* | 0 | 0 | 1 | 0 | 14 |
| *Engelhardia spicata var.* | 0 | 0 | 0 | 0 | 1 |
| *Eriolaena spectabilis* | 0 | 0 | 0 | 0 | 1 |
| *Eurya groffii* | 1 | 1 | 1 | 0 | 2 |
| *Ficus auriculata* | 1 | 0 | 1 | 0 | 4 |
| *Ficus cyrtophylla* | 0 | 0 | 0 | 0 | 1 |
| *Ficus esquiroliana* | 0 | 1 | 2 | 0 | 0 |
| *Ficus fistulosa* | 0 | 0 | 0 | 2 | 11 |
| *Ficus hirta* | 0 | 0 | 4 | 4 | 20 |
| *Ficus hispida* | 2 | 0 | 1 | 0 | 9 |
| *Ficus langkokensis* | 0 | 1 | 1 | 0 | 8 |
| *Ficus racemosa* | 0 | 0 | 2 | 0 | 0 |
| *Ficus sagittata* | 0 | 0 | 0 | 0 | 2 |
| *Ficus semicordata* | 3 | 1 | 6 | 0 | 3 |
| *Ficus vasculosa* | 0 | 0 | 1 | 0 | 1 |
| *Garcinia cowa* | 0 | 0 | 0 | 0 | 3 |
| *Garuga floribunda* | 0 | 0 | 0 | 0 | 1 |
| *Garuga pinnata* | 0 | 0 | 2 | 0 | 1 |
| *Gironniera subaequalis* | 0 | 0 | 0 | 0 | 22 |
| *Glochidion lanceolarium* | 0 | 0 | 0 | 0 | 2 |
| *Gmelina arborea* | 0 | 0 | 0 | 0 | 1 |
| *Gomphandra tetrandra* | 0 | 0 | 0 | 0 | 15 |
| *Harpullia cupanioides* | 0 | 0 | 0 | 0 | 6 |
| *Helicia nilagirica* | 0 | 0 | 0 | 0 | 1 |
| *Heliciopsis henryi* | 0 | 0 | 0 | 0 | 1 |
| *Hevea brasiliensis* | 0 | 0 | 5 | 3 | 1 |
| *Horsfieldia glabra* | 0 | 0 | 0 | 0 | 13 |
| *Ilex godajam* | 0 | 0 | 0 | 0 | 2 |
| *Knema furfuracea* | 0 | 0 | 0 | 0 | 29 |
| *Kydia calycina* | 0 | 1 | 0 | 0 | 11 |
| *Laurocerasus phaeosticta* | 0 | 0 | 0 | 0 | 24 |
| *Leea compactiflora* | 0 | 0 | 0 | 0 | 25 |
| *Linociera insignis* | 0 | 0 | 1 | 0 | 0 |
| *Lithocarpus fohaiensis* | 0 | 0 | 1 | 0 | 14 |
| *Lithocarpus truncatus* | 0 | 0 | 0 | 0 | 6 |
| *Litsea atrata* | 0 | 0 | 0 | 0 | 6 |
| *Litsea garrettii* | 0 | 0 | 0 | 0 | 1 |
| *Litsea glutinosa* | 0 | 3 | 3 | 1 | 3 |
| *Litsea monopetala* | 0 | 4 | 2 | 2 | 58 |
| *Litsea panamanja* | 0 | 0 | 0 | 0 | 2 |
| *Macaranga indica* | 0 | 2 | 4 | 0 | 2 |
| *Maclura pubescens* | 0 | 0 | 0 | 0 | 5 |
| *Macropanax dispermus* | 0 | 0 | 1 | 0 | 23 |
| *Maesa indica* | 0 | 0 | 0 | 0 | 2 |
| *Maesa montana* | 0 | 0 | 0 | 0 | 12 |
| *Mallotus barbatus* | 0 | 0 | 0 | 3 | 3 |
| *Mallotus paniculatus* | 0 | 5 | 0 | 0 | 2 |
| *Mallotus philippinensis* | 0 | 0 | 1 | 0 | 9 |
| *Mangifera indica* | 0 | 0 | 1 | 0 | 0 |
| *Markhamia stipulata* | 0 | 0 | 0 | 0 | 1 |
| *Mastixia pentandra* | 0 | 0 | 0 | 0 | 3 |
| *Mayodendron igneum* | 0 | 0 | 2 | 0 | 4 |
| *Medinilla septentrionalis* | 0 | 1 | 0 | 0 | 0 |
| *Meliosma arnottiana* | 0 | 0 | 0 | 0 | 15 |
| *Meliosma rigida* | 0 | 0 | 0 | 0 | 17 |
| *Memecylon cyanocarpum* | 0 | 0 | 0 | 0 | 7 |
| *Micromelum integerrimum* | 0 | 0 | 0 | 0 | 2 |
| *Morus macroura* | 0 | 0 | 0 | 0 | 27 |
| *Myristica yunnanensis* | 0 | 0 | 0 | 0 | 2 |
| *Nauclea officinalis* | 0 | 0 | 0 | 0 | 1 |
| *Neonauclea tsaiana* | 0 | 0 | 0 | 0 | 2 |
| *Nephelium chryseum* | 0 | 0 | 0 | 0 | 18 |
| *Olea rosea* | 0 | 0 | 0 | 0 | 2 |
| *Oroxylum indicum* | 0 | 0 | 0 | 0 | 7 |
| *Oxyceros sinensis* | 0 | 0 | 1 | 0 | 32 |
| *Paramichelia baillonii* | 0 | 0 | 0 | 0 | 3 |
| *Persea tenuipilis* | 0 | 0 | 0 | 0 | 10 |
| *Phoebe lanceolata* | 0 | 1 | 3 | 0 | 7 |
| *Phoebe puwenensis* | 0 | 0 | 0 | 0 | 4 |
| *Phyllanthus emblica* | 0 | 1 | 0 | 0 | 33 |
| *Pinanga discolor* | 0 | 0 | 0 | 0 | 8 |
| *Pittosporopsis kerrii* | 0 | 1 | 0 | 0 | 40 |
| *Polyalthia litseifolia* | 0 | 0 | 0 | 0 | 7 |
| *Pometia tomentosa* | 0 | 0 | 0 | 0 | 18 |
| *Pouteria grandifolia* | 0 | 0 | 0 | 0 | 3 |
| *Pterospermum lanceifolium* | 0 | 0 | 0 | 0 | 2 |
| *Pterospermum menglunense* | 0 | 0 | 0 | 0 | 11 |
| *Pygeum macrocarpum* | 0 | 0 | 0 | 0 | 1 |
| *Rhus chinensis* | 0 | 0 | 2 | 0 | 13 |
| *Sapium baccatum* | 0 | 0 | 1 | 0 | 3 |
| *Saprosma ternata* | 0 | 0 | 0 | 0 | 74 |
| *Sarcosperma kachinense* | 0 | 0 | 0 | 0 | 2 |
| *Saurauia napaulensis* | 1 | 0 | 0 | 0 | 1 |
| *Saurauia yunnanensis* | 0 | 0 | 0 | 0 | 4 |
| *Schefflera octophylla* | 0 | 0 | 0 | 0 | 3 |
| *Schima wallichii* | 0 | 0 | 7 | 0 | 21 |
| *Semecarpus reticulata* | 0 | 0 | 0 | 0 | 4 |
| *Sloanea tomentosa* | 0 | 0 | 0 | 0 | 1 |
| *Sterculia brevissima* | 0 | 0 | 0 | 0 | 3 |
| *Sterculia lanceolata* | 0 | 0 | 0 | 0 | 6 |
| *Stereospermum colais* | 0 | 1 | 4 | 0 | 5 |
| *Symplocos cochinchinensis* | 0 | 6 | 6 | 0 | 1 |
| *Syzygium forrestii* | 0 | 0 | 1 | 0 | 2 |
| *Syzygium oblatum* | 0 | 3 | 0 | 0 | 0 |
| *Tarennoidea wallichii* | 0 | 0 | 0 | 0 | 1 |
| *Toona ciliata* | 0 | 0 | 0 | 0 | 1 |
| *Trevesia palmata* | 0 | 0 | 1 | 0 | 1 |
| *Trichilia connaroides* | 0 | 0 | 2 | 0 | 5 |
| *Turpinia pomifera* | 0 | 0 | 0 | 0 | 3 |
| *Ulmus lanceifolia* | 0 | 0 | 0 | 0 | 5 |
| *Vernonia parishii* | 0 | 0 | 1 | 0 | 0 |
| *Vitex quinata* | 0 | 0 | 0 | 0 | 1 |
| *Wendlandia tinctoria subsp.* | 0 | 0 | 1 | 0 | 0 |
| *Wendlandia uvariifolia* | 0 | 0 | 0 | 0 | 7 |
| *Wrightia pubescens* | 0 | 0 | 2 | 0 | 1 |
| *Xanthophyllum siamense* | 0 | 0 | 0 | 0 | 7 |
| *Ziziphus rugosa* | 0 | 0 | 0 | 0 | 3 |
| Total species | 6 | 23 | 51 | 10 | 165 |
| Total individuals | 9 | 47 | 99 | 46 | 1233 |
