# Supplementary material for: Tree Diversity Mediates the Distribution of Longhorn Beetles (Coleoptera: Cerambycidae) in a Changing Tropical Landscape (Southern Yunnan, SW China)
Source: PLoS One. 2013 Sep 19;8(9):e75481. doi: 10.1371/journal.pone.0075481 (PMC3777904; doi:10.1371/journal.pone.0075481)
Supplement: Table S3 — List of longhorn beetle species and numbers of individuals recorded from the 13 study localities compiled by habitat types (*means indicator species). Abbreviation code FA, OP, RU and FO means rice fallow, open land, rubber plantation and forest respectively. (DOCX) [file pone.0075481.s003.docx]

**Supporting Information**

**Table S3** List of longhorn beetle species and numbers of individuals recorded from the 13 study localities compiled by habitat types (*means indicator species). Abbreviation code FA, OP, RU and FO means rice fallow, open land, rubber plantation and forest respectively.

| Species | FA | OP | RU(5-8y) | RU(20-40y) | FO |
| --- | --- | --- | --- | --- | --- |
| *Acalolepta cervina* (Hope, 1831) | 2 | 7 | 3 | 0 | 5 |
| *Acalolepta* spec. 1 | 1 | 0 | 0 | 0 | 0 |
| *Acalolepta* spec. 2 | 0 | 0 | 1 | 0 | 1 |
| *Aeolesthes sinensis* (Gahan, 1890) | 0 | 0 | 0 | 0 | 1 |
| *Aesopida malasiaca* (Thomson, 1864) | 0 | 1 | 0 | 0 | 2 |
| *Agelasta (Dissosira) mouhoti* (Pascoe, 1862) | 0 | 0 | 0 | 3 | 4 |
| *Agelasta (Pseudagelasta) birmanica* (Breuning, 1935) | 0 | 0 | 0 | 0 | 3 |
| *Alidus biplagiatus* (Gahan, 1893) | 0 | 0 | 0 | 0 | 3 |
| *Allotraeus (Nysina) orientalis* (White, 1853) | 0 | 0 | 0 | 0 | 1 |
| *Anastathes cf. nigricornis* (Thomson, 1865) | 0 | 0 | 0 | 0 | 3 |
| *Aphrodisium faldermanii* (Saunders, 1853) | 0 | 0 | 0 | 0 | 1 |
| *Apomecyna cretacea* (Hope, 1831) | 0 | 0 | 0 | 1 | 0 |
| *Apomecyna longicollis* (Pic, 1925) | 0 | 0 | 1 | 0 | 0 |
| *Apomecyna saltator* (Fabricius, 1781) | 0 | 1 | 0 | 0 | 2 |
| *Apomecyna tigrina* (Thomson, 1857) | 0 | 0 | 0 | 0 | 1 |
| *Arctolamia luteomaculata* (Pu, 1981) | 0 | 0 | 1 | 0 | 0 |
| *Artimpaza curtelineata* (Pic, 1922) | 0 | 1 | 0 | 0 | 2 |
| *Artimpaza mimetica* (Holzschuh, 1989) | 0 | 0 | 0 | 0 | 4 |
| *Cacia (Ipogregyes) cf. cephaloides* (Breuning, 1968) | 0 | 0 | 0 | 1 | 3 |
| *Capnolymma brunnea* (Gressitt & Rondon, 1970) | 0 | 1 | 0 | 0 | 0 |
| *Ceresium fallaciosum* (Holzschuh, 1995) | 0 | 0 | 1 | 1 | 6 |
| *Ceresium lepidulum* (Holzschuh, 1982) | 0 | 0 | 0 | 1 | 0 |
| *Ceresium nilgiriense* (Gahan, 1906) | 0 | 0 | 0 | 0 | 2 |
| *Ceresium* spec. 1 | 1 | 0 | 0 | 1 | 3 |
| *Ceresium* spec. 2 | 0 | 0 | 0 | 1 | 4 |
| *Chloridolum (Leontium) viride* (Thomson, 1864) | 0 | 1 | 0 | 0 | 1 |
| *Chlorophorus angustatus* (Pic, 1920) | 0 | 0 | 0 | 0 | 3 |
| *Chlorophorus annularis* (Fabricius, 1787) | 0 | 1 | 3 | 1 | 5 |
| *Chlorophorus annularoides* (Holzschuh, 1983) | 0 | 1 | 0 | 0 | 2 |
| *Chlorophorus annulatus* (Hope, 1831) | 18 | 13 | 1 | 2 | 9 |
| *Chlorophorus arciferus* (Chevrolat, 1863) | 0 | 0 | 11 | 9 | 25 |
| *Chlorophorus cf hainanensis* | 0 | 0 | 1 | 0 | 0 |
| *Chlorophorus cf. nepalensis* (Hayashi, 1979) | 0 | 0 | 0 | 1 | 3 |
| *Chlorophorus copiosus* (Holzschuh, 1991) | 0 | 1 | 0 | 4 | 0 |
| *Chlorophorus douei* (Chevrolat, 1863) | 1 | 3 | 7 | 4 | 11 |
| *Chlorophorus hederatus* (Heller, 1926) | 1 | 7 | 7 | 0 | 15 |
| *Chlorophorus ictericus* (Holzschuh, 1991) | 0 | 0 | 0 | 0 | 1 |
| *Chlorophorus inhumeralis* (Pic, 1918) | 0 | 1 | 1 | 0 | 1 |
| *Chlorophorus insidiosus* (Holzschuh, 1986) | 2 | 0 | 0 | 0 | 1 |
| *Chlorophorus quatuordecimmaculatus* (Chevrolat, 1863) | 0 | 0 | 2 | 0 | 9 |
| *Chlorophorus siegriedae* (Holzschuh, 1993) | 0 | 1 | 0 | 0 | 0 |
| *Chlorophorus* spec. 1 | 0 | 1 | 0 | 0 | 2 |
| *Chlorophorus* spec. 2 | 1 | 0 | 0 | 6 | 2 |
| *Chlorophorus* spec. 3 | 0 | 0 | 1 | 0 | 3 |
| *Chlorophorus* spec. 4 | 0 | 1 | 0 | 0 | 0 |
| *Cleomenes ornatus* (Holzschuh, 1981) | 0 | 0 | 0 | 0 | 1 |
| *Cleptometopus* spec. 1 | 1 | 0 | 0 | 0 | 6 |
| *Cleptometopus* spec. 2 | 0 | 2 | 0 | 0 | 2 |
| *Coptops annulipes* (Gahan, 1894) | 0 | 0 | 1 | 1 | 2 |
| *Coptops leucosticticus* (White, 1858) | 0 | 0 | 0 | 2 | 6 |
| *Demonax alcanor* (Gressitt & Rondon, 1970) | 0 | 0 | 1 | 0 | 0 |
| *Demonax annamensis* (Pic, 1943) | 0 | 1 | 0 | 0 | 1 |
| *Demonax dignus* (Gahan, 1894) | 0 | 3 | 1 | 1 | 2 |
| *Demonax elongatus* (Gressitt & Rondon, 1970) | 0 | 2 | 2 | 0 | 4 |
| *Demonax languidus* (Holzschuh, 1992) | 1 | 2 | 0 | 1 | 1 |
| *Demonax levipes* (Holzschuh, 1991) | 0 | 0 | 3 | 0 | 1 |
| *Demonax nansenensis* (Pic, 1903) | 0 | 0 | 0 | 0 | 1 |
| *Demonax nousophi* (Gressitt & Rondon, 1970) | 0 | 0 | 2 | 0 | 8 |
| *Demonax nr. inops* (Holzschuh, 1991) | 0 | 0 | 0 | 0 | 3 |
| *Demonax occultus* (Gressitt & Rondon, 1970) | 0 | 1 | 3 | 0 | 3 |
| *Demonax pumilio* (Holzschuh, 1991) | 0 | 0 | 1 | 1 | 2 |
| *Demonax reticulicollis* (Gressitt, 1940) | 0 | 0 | 0 | 0 | 2 |
| *Demonax semiluctuosus* (White, 1855) | 0 | 1 | 1 | 0 | 0 |
| *Demonax* spec. 1 | 0 | 0 | 1 | 0 | 9 |
| *Demonax testaceoannulatus* (Pic, 1935) | 0 | 0 | 0 | 0 | 5 |
| *Demonax trudae* (Holzschuh, 1983) | 0 | 0 | 0 | 0 | 10 |
| *Dere cf. femoralis* (Holzschuh, 1998) | 0 | 0 | 0 | 0 | 9 |
| *Desisa subfasciata* (Pascoe, 1862) | 0 | 0 | 0 | 0 | 2 |
| *Diastocera wallichi* (Hope, 1831) | 0 | 0 | 0 | 0 | 2 |
| *Dorysthenes (Lophosternus) buquetii* (Guèrin-Menevielle, 1844) | 0 | 0 | 0 | 0 | 5 |
| *Dymasius aureofulvescens* (Gressitt & Rondon, 1970) | 0 | 0 | 1 | 1 | 7 |
| *Egesina albolineata* (Breuning, 1943) | 0 | 2 | 0 | 0 | 2 |
| *Epepeotes luscus* (Fabricius, 1787) | 1 | 0 | 1 | 0 | 5 |
| *Epepeotes uncinatus* (Gahan, 1888)* | 0 | 0 | 0 | 0 | 5 |
| *Epiglenea comes* (Bates, 1884) | 0 | 0 | 0 | 0 | 5 |
| *Epipedocera laticollis* (Gahan, 1906) | 0 | 1 | 0 | 0 | 4 |
| *Epipedocera vitalisi* (Pic, 1922) | 0 | 1 | 0 | 0 | 2 |
| *Eucomatocera vittata* (White, 1846) | 0 | 1 | 1 | 0 | 7 |
| *Eunidia lateralis* (Gahan, 1893 det. M. Lin 2010) | 0 | 0 | 0 | 2 | 0 |
| *Euryphagus lundii* (Fabricius, 1792) | 0 | 5 | 4 | 0 | 10 |
| *Exocentrus alboguttatus* (Fisher, 1925) | 0 | 1 | 0 | 0 | 2 |
| *Exocentrus cf. constricticollis* (Gressitt, 1940) | 0 | 0 | 0 | 0 | 4 |
| *Falsomesosella gardneri* (Breuning, 1938) | 0 | 1 | 0 | 0 | 3 |
| *Glenea (Stiroglenea) cantor* (Fabricius, 1787) | 0 | 1 | 0 | 0 | 2 |
| *Glenea aeolis laosica* (Breuning, 1963) | 0 | 0 | 1 | 0 | 6 |
| *Glenea cancellata* (Thomson, 1865) | 0 | 0 | 0 | 0 | 2 |
| *Glenea cardinalis* (Thomson, 1860) | 0 | 0 | 0 | 0 | 1 |
| *Glenea citrinopubens* (Pic, 1926) stat. reinstated | 0 | 1 | 0 | 0 | 2 |
| *Glenea diana* (Thomson, 1865) | 0 | 0 | 0 | 0 | 5 |
| *Glenea diverselineata* (Pic, 1926) | 0 | 0 | 0 | 0 | 6 |
| *Glenea flavosignata* (Breuning, 1956) | 0 | 0 | 0 | 0 | 1 |
| *Glenea Indiana* (Thomson, 1857) | 0 | 0 | 0 | 1 | 1 |
| *Glenea langana* (Pic, 1903) stat. reinstated | 0 | 2 | 0 | 0 | 4 |
| *Glenea meiyingae* (Holzschuh, 2009) | 0 | 0 | 0 | 0 | 3 |
| *Glenea mouhoti* (Thomson, 1865) | 0 | 0 | 0 | 0 | 9 |
| *Glenea multiinterrupta* (Pic, 1947) stat. reinstated | 0 | 0 | 0 | 0 | 2 |
| *Glenea nigromaculata* (Thomson, 1865) | 0 | 1 | 0 | 0 | 0 |
| *Glenea pici* (Aurivillius, 1925) | 0 | 1 | 0 | 0 | 3 |
| *Glenea pulchra* (Aurivillius, 1926) | 0 | 0 | 1 | 0 | 1 |
| *Glenea quadrinotata* (Guérin-Méneville, 1843) | 0 | 0 | 0 | 0 | 8 |
| *Glenea siamensis* (Gahan, 1897) | 0 | 0 | 0 | 0 | 2 |
| *Glenea* sp.1 | 0 | 1 | 0 | 0 | 0 |
| *Glenea* sp.2 | 0 | 1 | 0 | 1 | 0 |
| *Glenea* sp.3 | 0 | 0 | 0 | 0 | 1 |
| *Glenea subviridescens* (Breuning, 1963) | 0 | 0 | 0 | 0 | 1 |
| *Glenea torquatella* (Aurivillius, 1923) | 0 | 0 | 0 | 0 | 3 |
| *Glenea vaga* (Thomson, 1865) | 1 | 0 | 0 | 0 | 2 |
| *Golsinda basicornis* (Gahan, 1894) | 0 | 0 | 0 | 0 | 2 |
| *Heteroglenea nigromaculata* (Thomson, 1865) | 3 | 12 | 17 | 0 | 9 |
| *Hirtaeschopalaea albolineata* (Pic, 1925) | 0 | 0 | 0 | 0 | 2 |
| *Imantocera penicillata* (Hope, 1831) | 0 | 2 | 0 | 0 | 3 |
| *Ischnodora sejugata* (Holzschuh, 1991) | 0 | 3 | 1 | 0 | 2 |
| *Macrochenus isabellinus* (Aurivillius, 1920) | 0 | 0 | 1 | 0 | 1 |
| *Menesia niveoguttata* (Aurivillius, 1925) | 0 | 0 | 2 | 2 | 0 |
| *Merionoeda (Macromolorchus) baoshana* (Chiang, 1963) | 0 | 0 | 1 | 0 | 3 |
| *Mesocacia multimaculata* (Pic, 1925) | 0 | 0 | 0 | 2 | 2 |
| *Mesosa (Aphelocnemia) subfasciata* (Gahan, 1894) | 0 | 0 | 1 | 0 | 3 |
| *Mesosa nigrofasciaticollis* (Breuning, 1968) | 0 | 0 | 1 | 0 | 2 |
| *Mesosa obscura* (Gahan, 1894) | 0 | 0 | 1 | 1 | 1 |
| *Microdebilissa infirma* (Holzschuh, 1989) | 0 | 0 | 0 | 1 | 4 |
| *Mispila punctifrons* (Breuning, 1938) | 0 | 0 | 0 | 0 | 1 |
| *Moechotypa asiatica* (Pic, 1903) | 1 | 2 | 4 | 0 | 3 |
| *Moechotypa delicatula* (White, 1858)* | 0 | 0 | 6 | 0 | 22 |
| *Monochamus* (?) spec. | 0 | 0 | 0 | 0 | 4 |
| *Monochamus bimaculatus* (Gahan, 1888) | 0 | 8 | 1 | 1 | 5 |
| *Monochamus dubius* (Gahan, 1894) | 0 | 0 | 0 | 1 | 1 |
| *Monochamus guerryi* (Pic, 1903) | 0 | 1 | 0 | 4 | 0 |
| *Mycerinopsis lineata* (Gahan, 1894) | 0 | 3 | 2 | 2 | 2 |
| *Nida flavovittata* (Pascoe, 1867) | 1 | 9 | 2 | 2 | 5 |
| *Niphona falaizei* (Breuning, 1962) | 0 | 0 | 1 | 0 | 4 |
| *Nupserha ambigua* (Lameere, 1893) | 9 | 5 | 0 | 0 | 5 |
| *Nupserha cf. yunnanensis* (Breuning, 1960) | 0 | 1 | 0 | 0 | 5 |
| *Nupserha clypealis* (Fairmaire, 1895) | 4 | 0 | 5 | 1 | 3 |
| *Nupserha dubia* (Gahan, 1884) | 0 | 2 | 0 | 0 | 1 |
| *Nupserha fricator* (Dalman, 1817) | 1 | 0 | 0 | 0 | 3 |
| *Nupserha lenita* (Pascoe, 1867) | 0 | 9 | 0 | 1 | 4 |
| *Nupserha minor* (Pic, 1939) | 5 | 11 | 7 | 0 | 2 |
| *Nupserha multimaculata* (Pic, 1939) | 0 | 1 | 0 | 0 | 1 |
| *Nupserha nigriceps* (Gahan, 1894) | 2 | 5 | 24 | 1 | 6 |
| *Nupserha* spec.1 | 0 | 0 | 1 | 0 | 4 |
| *Nupserha variabilis* (Gahan, 1894) | 0 | 0 | 0 | 2 | 1 |
| *Nupserha ventralis* (Gahan, 1894) | 2 | 1 | 0 | 0 | 2 |
| *Nyctimenius tristis* (Fabricius, 1792) | 0 | 2 | 0 | 0 | 2 |
| *Oberea birmanica* (Gahan, 1894) | 0 | 1 | 2 | 0 | 1 |
| *Oberea cf. consentanea* (Pascoe, 1867) | 0 | 0 | 0 | 0 | 5 |
| *Oberea clara* (Pascoe, 1866) | 3 | 2 | 1 | 0 | 4 |
| *Oberea ferrugine*a (Thunberg, 1787) | 0 | 0 | 0 | 0 | 1 |
| *Oberea lacana* (Pic, 1923) | 0 | 0 | 0 | 0 | 5 |
| *Oberea laosensis* (Breuning, 1963) | 0 | 0 | 0 | 0 | 6 |
| *Oberea cf. birmanica* (Gahan, 1895) | 0 | 1 | 2 | 0 | 2 |
| *Obereopsis cf. modica* (Gahan, 1894) | 0 | 1 | 1 | 0 | 3 |
| *Oemospila* (?) *callidioides* (Gressitt & Rondon, 1970) | 0 | 0 | 0 | 0 | 1 |
| *Olenecamptus bilobus* (Fabricius, 1801) | 0 | 0 | 0 | 0 | 5 |
| *Ostedes* spec. 1 | 0 | 0 | 0 | 1 | 4 |
| Paraleprodera insidiosa (Gahan, 1888) | 0 | 0 | 0 | 1 | 9 |
| Parasalpinia laosensis (Gressitt & Rondon, 1970) | 0 | 0 | 0 | 0 | 4 |
| *Perissus aemulus* (Pascoe, 1869) | 0 | 2 | 2 | 0 | 8 |
| *Perissus cf. mimicus* (Gressitt & Rondon, 1970) | 1 | 2 | 2 | 2 | 8 |
| *Perissus mutabilis* (Gahan, 1894) | 1 | 0 | 0 | 0 | 6 |
| *Perissus persimilis* (Gahan, 1894) | 1 | 1 | 0 | 0 | 0 |
| *Pharsalia (Cycos) subgemmata* (Thomson, 1857) | 0 | 0 | 2 | 0 | 11 |
| *Phelipara marmorata* (Pascoe, 1866) | 0 | 0 | 1 | 0 | 1 |
| *Pothyne rugifrons* (Gressitt, 1940) | 1 | 0 | 0 | 0 | 1 |
| *Pothyne variegata* (Thomson, 1864) | 0 | 0 | 0 | 1 | 0 |
| *Prothema auratum* (Gahan, 1906) | 0 | 0 | 0 | 0 | 2 |
| *Pseudocalamobius* spec.1 | 0 | 1 | 0 | 1 | 4 |
| *Pterolophia (Hylobrotus) lateralis* (Gahan, 1894) | 0 | 1 | 1 | 0 | 3 |
| *Pterolophia alboplagiata* (Gahan, 1894) | 0 | 2 | 2 | 0 | 2 |
| *Pterolophia annulata* (Chevrolat, 1845)* | 0 | 0 | 9 | 5 | 26 |
| *Pterolophia cf. melanura* (Pascoe, 1857) | 3 | 6 | 23 | 3 | 26 |
| *Pterolophia consularis* (Pascoe, 1866) | 0 | 3 | 0 | 0 | 4 |
| *Pterolophia externemaculata* (Breuning, 1938) | 0 | 0 | 1 | 0 | 0 |
| *Pterolophia lunigera* (Aurivillius, 1913) | 0 | 1 | 2 | 0 | 1 |
| *Pterolophia multifasciculata* (Pic, 1926) | 0 | 1 | 1 | 0 | 0 |
| *Pterolophia nigrofasciculata* (Breuning, 1938) | 0 | 0 | 0 | 0 | 1 |
| *Pterolophia nr. jeanvoinei* (Pic, 1929) | 1 | 0 | 1 | 0 | 4 |
| *Pterolophia persimilis* (Gahan, 1894) | 0 | 0 | 0 | 0 | 7 |
| *Rhaphuma aequalis* (Holzschuh, 1991) | 0 | 0 | 0 | 0 | 4 |
| *Rhaphuma anongae* (Gressit & Rondon, 1970) | 0 | 0 | 3 | 4 | 4 |
| *Rhaphuma bicolorifemoralis* (Gressitt & Rondon, 1970) | 0 | 0 | 2 | 0 | 3 |
| *Rhaphuma cf.binhensis maculicollis* (Gressitt & Rondon, 1970) | 0 | 0 | 0 | 0 | 1 |
| *Rhaphuma clarina* (Gressitt & Rondon, 1970) | 0 | 0 | 2 | 0 | 2 |
| *Rhaphuma constricta* (Gressitt & Rondon, 1970) | 0 | 1 | 0 | 0 | 1 |
| *Rhaphuma desaii* (Gardner, 1940) | 0 | 0 | 0 | 0 | 1 |
| *Rhaphuma diana* (Gahan, 1906) | 0 | 1 | 0 | 0 | 1 |
| *Rhaphuma elongata* (Gressitt, 1940) | 0 | 0 | 1 | 0 | 0 |
| *Rhaphuma encausta* (Holzschuh, 1991) | 1 | 0 | 0 | 0 | 0 |
| *Rhaphuma falx* (Holzschuh, 1991) | 0 | 0 | 0 | 1 | 2 |
| *Rhaphuma illicata* (Holzschuh, 1991)* | 0 | 0 | 0 | 0 | 7 |
| *Rhaphuma laosica* (Gressitt & Rondon, 1970) | 0 | 0 | 0 | 0 | 4 |
| *Rhaphuma maculicollis* (Gressitt & Rondon, 1970) | 0 | 0 | 0 | 1 | 4 |
| *Rhaphuma nr. horsfieldi* (White, 1855) | 0 | 0 | 0 | 1 | 1 |
| *Rhaphuma paucis* (Holzschuh, 1992) | 0 | 0 | 0 | 0 | 1 |
| *Rhaphuma* *nr. incarinata* (Pic, 1925) | 0 | 0 | 0 | 0 | 20 |
| *Rhytidodera bowringii* (White, 1853) | 0 | 0 | 0 | 0 | 4 |
| *Rhytidodera integra* (Kolbe, 1886) | 0 | 0 | 0 | 1 | 1 |
| *Rondibilis paralineaticollis* (Breuning, 1968)* | 0 | 0 | 0 | 0 | 7 |
| *Ropica* spec. 1 | 0 | 0 | 1 | 1 | 0 |
| *Ropica* spec. 2 | 0 | 0 | 0 | 0 | 1 |
| *Sarmydus loebli* (Drumont & Weigel, 2010) | 0 | 1 | 0 | 0 | 1 |
| *Serixia pubescens* (Gressitt, 1940) | 0 | 1 | 1 | 0 | 3 |
| *Similosodus cf. choumi* (Breuning, 1962) | 0 | 0 | 0 | 0 | 3 |
| *Sophronica cf. atripennis* (Pic, 1926) | 0 | 0 | 0 | 1 | 2 |
| *Stenhomalus odai* (Niisato & Kinugasa, 1982) | 0 | 0 | 0 | 0 | 1 |
| *Stenodryas atripes* (Pic, 1935) | 0 | 0 | 0 | 0 | 2 |
| *Sthenias fransiscanus* (Thomson, 1865) | 0 | 0 | 0 | 1 | 4 |
| *Stibara rufina* (Pascoe, 1858) | 0 | 2 | 1 | 0 | 0 |
| *Tetraglenes hirticornis* (Fabricius, 1798) | 0 | 2 | 9 | 0 | 2 |
| *Thranius multinotatus* (Pic, 1922) | 0 | 1 | 0 | 0 | 0 |
| *Trypogeus aureopubens* (Pic, 1903) | 0 | 1 | 0 | 0 | 1 |
| *Uraecha punctata* (Gahan, 1888) | 0 | 1 | 2 | 1 | 5 |
| *Uraecha* spec.1 | 0 | 0 | 0 | 0 | 3 |
| *Xoanodera maculata* (Schwarzer, 1925) | 1 | 0 | 1 | 0 | 4 |
| *Xoanodera regularis* (Gahan, 1890) | 0 | 1 | 0 | 5 | 2 |
| *Xylotrechus buqueti* (Laporte & Gory, 1836)* | 1 | 11 | 14 | 5 | 26 |
| *Xylotrechus javanicus* (Laporte & Gory, 1836) | 0 | 2 | 2 | 0 | 6 |
| *Xylotrechus tanoni* (Gressitt & Rondon, 1970) | 0 | 0 | 0 | 0 | 4 |
| *Zotalemimon ciliatum* (Gressitt, 1942) | 2 | 1 | 0 | 2 | 1 |
| *Zotalemimon costatum* (Matsushita,1933) | 1 | 2 | 2 | 0 | 1 |
| *Zotalemimon* spec.1 | 0 | 0 | 0 | 0 | 3 |
| Total species | 33 | 82 | 77 | 55 | 193 |
| Total individuals | 76 | 204 | 238 | 106 | 780 |
